# Allosteric inhibition of TEM‐1 β lactamase: Microsecond molecular dynamics simulations provide mechanistic insights

**DOI:** 10.1002/pro.4622

**Published:** 2023-04-01

**Authors:** Erich Hellemann, Amrita Nallathambi, Jacob D. Durrant

**Affiliations:** ^1^ Department of Biological Sciences University of Pittsburgh Pittsburgh Pennsylvania 15260 USA

**Keywords:** allostery, antibiotics, molecular dynamics simulations, TEM‐1

## Abstract

β‐lactam antibiotics target DD‐transpeptidases, enzymes that perform the last step of bacterial cell‐wall synthesis. To block the antimicrobial activity of these antibiotics, bacteria have evolved lactamases that render them inert. Among these, TEM‐1, a class A lactamase, has been extensively studied. In 2004, Horn et al. described a novel allosteric TEM‐1 inhibitor, FTA, that binds distant from the TEM‐1 orthosteric (penicillin‐binding) pocket. TEM‐1 has subsequently become a model for the study of allostery. In the present work, we perform molecular dynamics simulations of FTA‐bound and FTA‐absent TEM‐1, totaling ~3 μS, that provide new insight into TEM‐1 inhibition. In one of the simulations, bound FTA assumed a conformation different than that observed crystallographically. We provide evidence that the alternate pose is physiologically plausible and describe how it impacts our understanding of TEM‐1 allostery.

## 1 INTRODUCTION

The discovery of antibiotics in the early 20th century has drastically reduced morbidity and mortality. However, decades of indiscriminate prophylactic and therapeutic use in agriculture, medicine, and research have put tremendous selective pressure on bacteria, forcing them to evolve resistance (Blair et al., 2015; Davies & Davies, 2010; Zaffiri et al., 2012). The World Health Organization (WHO) recognizes antimicrobial resistance as one of the top 10 global public health threats facing humanity (WHO, 2021).

β‐lactam antibiotics, the most prescribed class of antibiotics (Tooke et al., 2019), target DD‐transpeptidases (i.e., penicillin‐binding proteins, PBPs), enzymes that perform the last step of bacterial cell‐wall synthesis by cross‐linking peptides, thus completing peptidoglycan synthesis. To block the antimicrobial activity of β‐lactam antibiotics, bacteria have evolved lactamases that render these compounds inert by opening their lactam rings via hydrolysis. Lactamases are divided into four classes; class A, B, and C lactamases are serine hydrolases, and class D lactamases are metallo‐lactamases.

TEM‐1, a class A lactamase, was first identified in 1963 in bacteria resistant to penicillin (Datta & Richmond, 1966). Its discovery prompted the development of new, less susceptible lactam antibiotics, which inevitably provoked the evolution of new lactamases (i.e., the “β‐lactamase cycle”) (Matagne et al., 1999; Salverda et al., 2010). There are currently over 240 known TEM variants, as reported in the Beta‐Lactamase DataBase (Naas et al., 2017).

**FIGURE 1:**
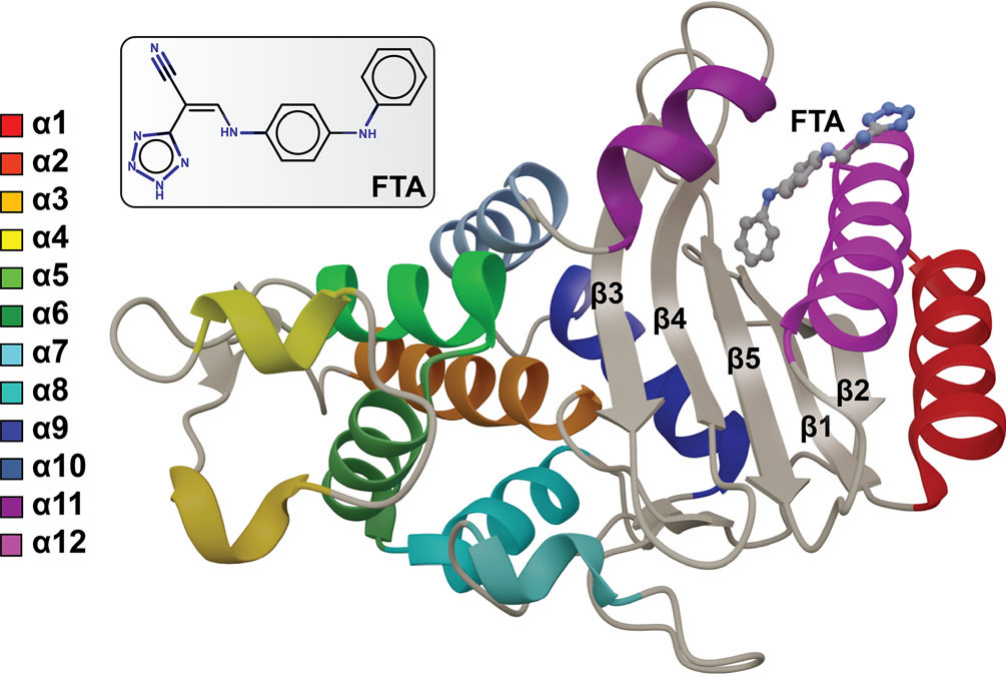
TEM‐1 β‐lactamase bound to the allosteric inhibitor FTA. The protein structure is taken from PDB 1PZP. Helices are color coded, and sheets are labeled. The FTA schematic was generated using MarvinSketch 18.24.0, ChemAxon, www.chemaxon.com.

Class A β‐lactamases have two tightly packed domains (a primarily α‐helical domain and an α/β‐domain) that are connected by two hinges (Figure 1). The mostly α‐helical domain contains nine helices and includes most of the residues that line the catalytic, penicillin‐binding site. The α/β‐domain, which contains three α‐helices and five anti‐parallel β‐sheets, includes a cryptic allosteric site that forms when α11 rotates and moves away from α12 (Horn & Shoichet, 2004).

In the present work, we perform molecular dynamics (MD) simulations of *holo* and *apo* TEM‐1 totaling ~3 μS. The *holo* simulations include the allosteric inhibitor 3‐(4‐phenylamino‐phenylamino)‐2‐(1H‐tetrazol‐5‐yl)‐acrylonitrile (FTA), which binds in the cryptic pocket (Horn & Shoichet, 2004) (Figure 1). To capture the impact of this allosteric ligand on protein dynamics before penicillin binding, we leave the orthosteric pocket unoccupied. These simulations suggest that FTA binding alters the dynamics of the R244 side chain, moving it away from the orthosteric pocket where β‐lactam antibiotics bind. This shift is important because R244 stabilizes bound β‐lactam antibiotics via an interaction with their carboxylate groups (Horn & Shoichet, 2004). The altered R244 dynamics may therefore weaken orthosteric binding, preventing inactivation via hydrolysis. FTA also changes inter‐domain protein communication, which could further impact inhibition.

In one of our *holo* simulations, the FTA‐bound allosteric pocket opens considerably, adopting a conformation similar to that reported when TEM‐1 is bound to N,N‐bis(4‐chlorobenzyl)‐1H‐1,2,3,4‐tetrazol‐5‐amine (PDB 1PZO) (Horn & Shoichet, 2004). This conformational transition allows the bound FTA to undergo substantial rearrangement, adopting a new pose that differs from the pose captured crystallographically. We discuss whether this second pose is physiological and raise the possibility that the crystallographic pose may be artefactual, given the presence of crystal contacts. Regardless, we are hopeful that the alternative pocket conformation will aid medicinal chemists in the design of novel TEM inhibitors.

## 2 RESULTS AND DISCUSSION

To examine how the allosteric inhibitor FTA impacts the TEM‐1 orthosteric (catalytic) pocket to discourage the binding of β‐lactam antibiotics, we performed molecular dynamics (MD) simulations of *apo* (ligand‐absent) and *holo* (FTA‐bound) TEM‐1. The *apo* system included no small‐molecule ligands (i.e., neither FTA nor an orthosteric β‐lactam antibiotic). The *holo* system included FTA bound in the allosteric pocket, but no β‐lactam antibiotic. We ran three replicates of each equilibrated system in the NPT ensemble (1 μs of simulation time per system).

### 2.1 FTA binding alters TEM‐1 dynamics

To better understand how FTA binding impacts protein flexibility, we calculated the per‐residue root mean square fluctuations (RMSF) of the *holo* and *apo* systems, using each residue's center of geometry (Table S1). Overall, we found that FTA binding reduces TEM‐1 flexibility: ${\left\langle \text{RMSF}\right\rangle }_{\text{holo}}=0.71\pm 0.32\;Å$, and ${\left\langle \text{RMSF}\right\rangle }_{\mathrm{apo}}=0.78\pm 0.39\;Å$. Previous simulations of TEM‐1 bound to BLIP, a protein known to inhibit TEM‐1, also found that the inhibitor‐bound systems had reduced mean square fluctuations (Huang et al., 2020; Meneksedag et al., 2013).

**FIGURE 2:**
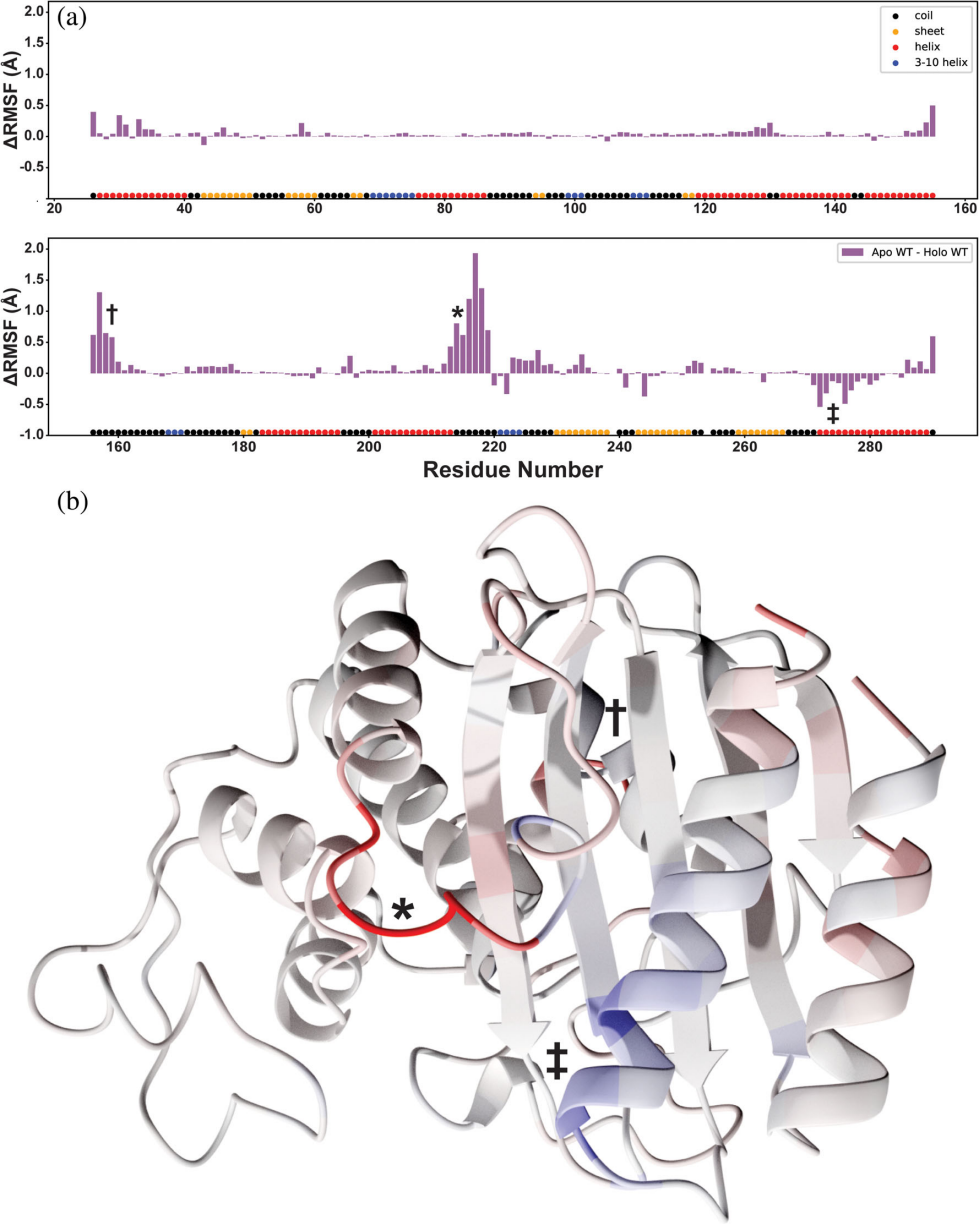
FTA binding decreases protein flexibility on average. (a) Differences in per‐residue RMSF (residue center of geometry), *apo* − *holo* simulations. When FTA is bound, two regions have reduced flexibility (marked with * and †), and one has increased flexibility (marked with ‡). See Table S1 for complete data, including standard errors of the mean for each system. (b) The same ΔRMSF values (*apo* − *holo*) per residue, projected onto the first frame of the *apo* simulation. Residues with higher *apo* RMSF values are shown in red, and residues with higher *holo* RMSF values are shown in blue. The same three regions marked in panel A are also indicated with *, †, and ‡. The region marked † is located on the back side of the protein from this angle and so is largely obscured.

To identify which specific TEM‐1 regions are most impacted by FTA binding, we calculated ΔRMSF values (*apo* − *holo*) per residue (Figure 2 and Figure S2) (Huang et al., 2020; Meneksedag et al., 2013). Binding substantially reduces the flexibility of two regions (Figure 2b, in red). The first is a hinge region comprised of the loop connecting helices α10 and α11 (residues A213‐G218, marked *). The α11 helix has direct contacts with the allosteric ligand FTA, and the A213‐G218 loop lines the orthosteric (penicillin‐binding) pocket, suggesting these altered dynamics may contribute to FTA‐mediated changes in penicillin binding.

The second region with reduced flexibility when FTA is bound spans residues M155‐L162, on the opposite side of the protein from the FTA‐ and penicillin‐binding pockets (Figure 2, marked †). Given that this region is distant from both binding pockets, it is unclear what role, if any, it plays in the allosteric mechanism. Future studies using mutagenesis (whether experimental or in silico) could potentially provide further mechanistic insight.

Although the *holo* (FTA‐bound) simulations are less flexible than the *apo* simulations on average (per RMSF), the α12 N‐terminus (Q269‐I279, Figure 2, marked ‡) is somewhat more flexible when FTA is bound (Figure 2b, in blue). The C‐terminus of this helix contacts FTA, and one of its residues, M272, comes within 7 Å of bound penicillin (per PDBs 1PZP (Horn & Shoichet, 2004) and 1FQG (Strynadka et al., 1992) when aligned), again suggesting that altered dynamics may contribute to FTA‐mediated changes in penicillin binding.

Curiously, recent simulations performed by Haider et al. suggest *holo* (FTA‐bound) TEM‐1 is more flexible than *apo* TEM‐1 (Galdadas et al., 2021). Specifically, they found that residues A213‐A224, which include the region marked * in Figure 2, are more flexible in the *holo* state; our simulations do not corroborate this result. The Haider simulations also suggest that FTA binding increases the flexibility of residues M155‐W165 (roughly the same as the region marked † in Figure 2); in contrast, we find that FTA reduces the flexibility of these residues. Finally, Haider et al. suggest that residues G196‐T200 are more flexible in the *holo* protein, but we detect no substantial difference in flexibility (Figure 2a).

The Haider simulations differ from ours methodologically in several ways, perhaps explaining the discrepancy. First, they created their *apo* TEM‐1 system by deleting FTA from a *holo* structure (PDB 1PZP; Horn & Shoichet, 2004) and equilibrating the resulting perturbed protein; in contrast, our *apo* simulations are derived directly from an *apo* crystal structure (PDB 1ZG4; Stec et al., 2005). Second, their TEM‐1 system differed from ours by five amino acids because they built systems based on the 1PZP (Horn & Shoichet, 2004) sequence (not the 1ZG4 (Stec et al., 2005) sequence) and removed two unstructured residues from the TEM‐1 C‐terminus. They also ran their simulations in the canonical ensemble (NVT), but we ran in the isothermal–isobaric ensemble (NPT). Other minor differences in the protocol (e.g., using RESP vs. AM1‐BCC to calculate partial atomic charges, using the ACEMD (Doerr et al., 2016) vs. the NAMD (Phillips et al., 2020) MD engine, etc.) may also account for the difference (Galdadas et al., 2021).

### 2.2 Holo simulation suggests an alternate FTA pose

**FIGURE 3:**
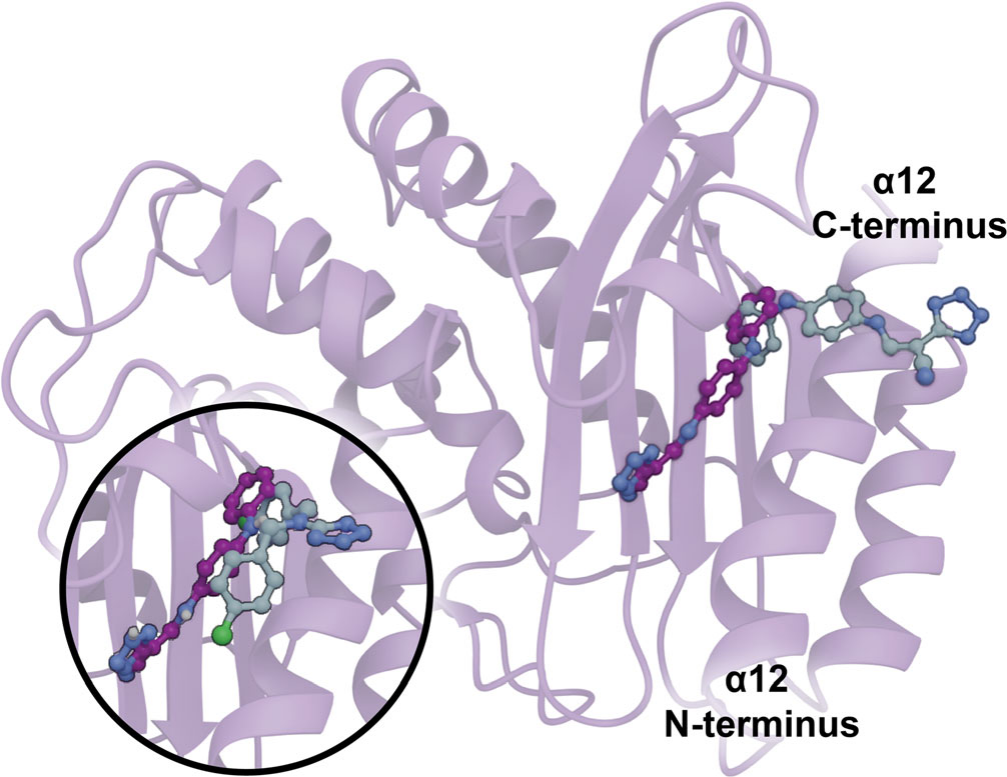
Allosteric ligand binding poses. Main figure: the vertical (PDB 1PZP) and horizontal FTA poses are shown in gray‐ and purple‐carbon representation, respectively. Inset figure: the crystallographic pose of CBT (PDB 1PZO) and the horizontal FTA pose are shown in gray‐ and purple‐carbon representation, respectively.

In all three *holo* simulations, the initial FTA pose was similar to that seen in PDB 1PZP (Horn & Shoichet, 2004). We call this pose the “vertical” pose because the FTA tetrazole moiety protrudes from the allosteric site perpendicular to the protein surface (Figure 3).

Surprisingly, FTA underwent substantial rearrangement in one of the three *holo* production runs, burying itself between α11 and α12 (Figure 3). The secondary amine bridging FTA's two aromatic rings initially points towards the α12 N‐terminus and interacts with the I279 carbonyl oxygen atom. When this interaction breaks, the amine inverts to point toward the α12 C‐terminus. The L221 side chain then packs closer to the β‐sheet, further opening the pocket, and α12 rotates counterclockwise. Finally, the tetrazole moiety inserts between α11 and α12. We call this inserted pose the “horizontal” pose (Figure 3, dark purple).

To rationalize this FTA insertion, we further analyzed the vertical FTA pose of the 1PZP *holo* structure (Horn & Shoichet, 2004). The electron density maps associated with this structure reveal that FTA forms some crystal contacts, including a hydrogen bond between one of the tetrazole nitrogen atoms and K192 of the neighboring protein in the crystal lattice (distance 3.1 Å between heavy atoms). This crystal packing contact could artefactually stabilize the vertical pose. Indeed, the 1PZP structure was resolved by soaking TEM‐1 crystals with FTA, a method that is known to sometimes yield different poses than those obtained via the slower cocrystallization method (Wienen‐Schmidt et al., 2021).

Several previous studies provide some support for the horizontal FTA pose we observed in our *holo* simulation. The first describes a TEM‐1 crystal structure (PDB 1PZO; Horn & Shoichet, 2004) that includes two copies of the ligand N,N‐bis(4‐chlorobenzyl)‐1H‐1,2,3,4‐tetrazole‐5‐amine (CBT). Curiously, the tetrazole ring of one of these copies is positioned near the horizontal‐pose FTA tetrazole, giving some experimental credence to the alternate pose (Figure 3, inset). A second study used elastic network models to assess allosteric‐pocket opening. This work predicted an open‐pocket conformation that may be able to accommodate the ligand in the horizontal position (Kaynak et al., 2020). Finally, two studies using MD simulations and Markov state models found evidence that the allosteric site opens further than even the ligand‐bound crystal structures suggest (Porter et al., 2019; Bowman & Geissler, 2012). This prior work supports the pocket flexibility we observed in our simulations, which enabled the observed FTA rearrangement.

### 2.3 The horizontal ligand pose is plausible per computational assessment

To determine whether the horizontal ligand pose observed in silico is plausible, we followed a pose‐assessment protocol described previously (Clark et al., 2016), with only minor modifications. First, we generated a four‐member ensemble of protein/ligand complexes that represented the horizontal and vertical poses equally. With this ensemble, we aimed to account for subtle but impactful conformational differences that distinguish otherwise similar protein/ligand complexes. The ensemble included two vertical‐pose conformations. One was taken from the *holo* simulation that captured the FTA pose transition; we clustered all vertical‐pose frames and extracted the centroid of the most‐populated cluster. The other was the crystallographic pose (PDB 1PZP; Horn & Shoichet, 2004). The ensemble also included two horizontal‐pose conformations taken from the *holo* simulation. One was identified using clustering as above, and the other was hand selected from a portion of the simulation where the ligand was completely embedded in the pocket and stable.

We used Schrödinger Maestro's induced‐fit docking (IFD) module (Clark et al., 2016; Sherman et al., 2006a, 2006b) to further expand the conformational ensemble. Unlike standard docking protocols, IFD accounts for the flexibility of the protein, albeit at the cost of computation time. We redocked FTA back into each of the four ensemble conformations and considered the top 15 protein/ligand conformations resulting from each redocking. Redocking FTA into the vertical‐pose conformations produced only vertically posed ligands, and redocking FTA into the horizontal‐pose conformations produced only horizontally posed ligands. This expanded ensemble therefore included 30 conformational variants of FTA in the vertical pose, and 30 with FTA in the horizontal pose. The IFDScores of the top‐ranking horizontal‐ and vertical‐pose ligands were comparable (−11,554.00 vs. −11,653.68, respectively; Table 1), suggesting both poses are plausible.

**TABLE 1:** Ligand‐pose assessment using induced‐fit docking (IFD), MM‐GBSA, and binding‐pose metadynamics (BPMD).

Pose	Best IFDScore	Best MM‐GBSA (kcal/mol)	BPMD (CompScore)
Vertical	−11,653.68	−63.56	2.72
Horizontal	−11,554.00	−81.35	1.26

*Note*: IFD was used to redock FTA into two vertical‐pose pocket conformations, one taken from a crystal structure (1PZP), and one taken from the MD simulation (identified using clustering). IFD was also used to redock FTA into two distinct horizontal‐pose conformations, both extracted from the simulation. We considered the top 15 predicted poses from each IFD run (60 total). MM‐GBSA was used to further assess the predicted IFD poses. The single best‐predicted vertical and horizontal poses per MM‐GBSA were further assessed using BPMD.

We next used Maestro's MM‐GBSA module(Schrödinger Maestro, 2021) to estimate the binding affinities of the 60 protein/ligand conformations. MM‐GBSA is a simulation‐based method that is typically more accurate than docking scoring functions (Guimaraes & Cardozo, 2008; Wang et al., 2019). We found that the top‐ranking horizontal pose had a better predicted free energy of binding than the top‐ranking vertical pose (−81.35 kcal/mol vs. ‐64.00 kcal/mol; Table 1).

Binding‐pose stability is another effective way to assess pose plausibility. Compounds that are incorrectly posed often slip in the binding pocket, but correctly posed ligands tend to remain stable (Sherman et al., 2006a, 2006b). We used Maestro's binding pose metadynamics (BPMD) to assess the stability of the single best‐ranking (per MM‐GBSA) horizontal and vertical pose, respectively. Given a simulation of a ligand pose, BPMD calculates a CompositeScore (CompScore) that accounts for (1) how much the ligand drifts from its original position and (2) the persistence of protein/ligand hydrogen bonds. Lower BPMD values correspond to more stable protein/ligand complexes (Allegra et al., 2021). Our BPMD calculations show higher stability for the horizontal pose (1.26) compared to the vertical pose (2.72, Table 1). For the sake of comparison, we also performed BPMD on a crystallographic (vertical) pose (PDB 1PZP; Horn & Shoichet, 2004) and the hand‐selected horizontal pose mentioned above, and saw similar trends (3.04 vs. 1.65).

These in silico results suggest the horizontal FTA pose is at least plausible. The IFDScores of the two poses are comparable, and the best‐scoring horizontal pose has better predicted free energy of binding (per MM‐GBSA) and stability (per BPMD).

### 2.4 FTA may impact β‐lactam‐antibiotic binding via altered Y105 dynamics

To understand how FTA‐induced changes in side‐chain dynamics contribute to the allosteric mechanism, we considered seven critical residues that are responsible for ligand recognition, ligand stabilization, or catalysis (Horn & Shoichet, 2004; Doucet et al., 2004; Kalp et al., 2009): K73, Y105, E166, W229, K234, R244, and R275. For each of these residues, we calculated Janin plots (Janin & Wodak, 1978) to characterize side‐chain dynamics (Figure S3). A Janin plot is similar to a Ramachandran plot, except it compares the first and second dihedral angles of the side chain (*χ*_1_ vs. *χ*_2_) instead of the dihedrals of the backbone (*φ* vs. *ψ*).

**FIGURE 4:**
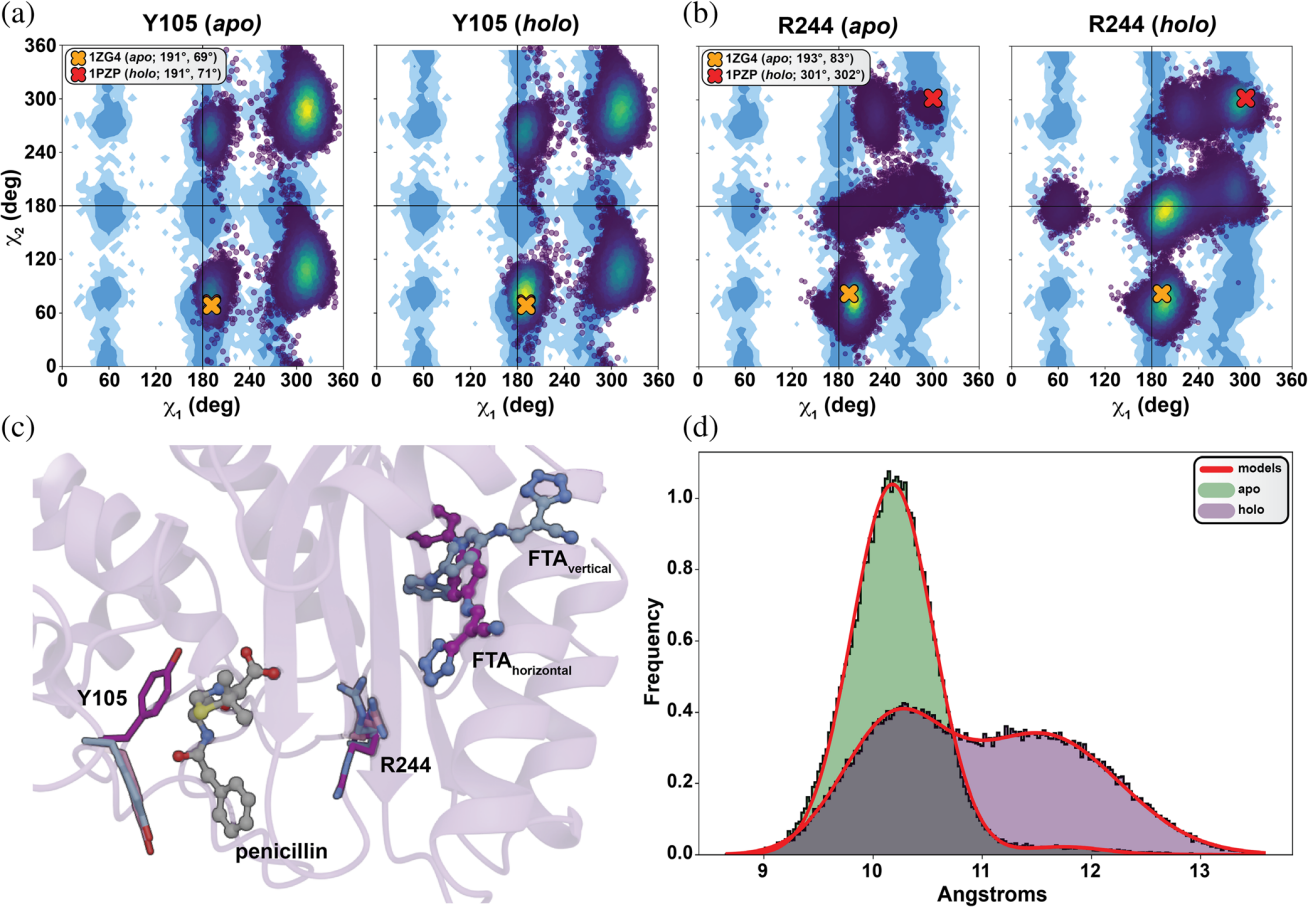
FTA binding influences Y105 and R244 dynamics. (a) Y105 Janin plots for the *apo* and *holo* simulations. Values in parentheses indicate the *χ*_1_ and *χ*_2_ values, in degrees. (b) R244 Janin plots for the *apo* and *holo* simulations. (c) Representative conformations of the Y105 and R244 side chains. FTA in the vertical and horizontal pose, as well as penicillin from the 1FQG structure, are superimposed for reference. (d) Distance distributions between R244 Cζ and S70 Cα.

This analysis suggested the dynamics of the Y105 sidechain (Figure 4) were substantially impacted by FTA binding. Y105, an amino acid that lines the catalytic pocket, is crucial for ligand recognition (Doucet et al., 2004). Our simulations reveal that its side chain adopts two main states (Figure 4a,c and Figure S3). In the first, the Y105 phenolic ring points towards the solvent and away from the orthosteric pocket, a conformation also observed in structures of TEM‐1 bound to cephalexin (PDB 4ZJ1 (Xiao et al., 2015), 4ZJ2 (Xiao et al., 2015), and 4ZJ3 (Xiao et al., 2015)) and the protein inhibitor BLIP (Lim et al., 2001) (PDB 2B5R; Reichmann et al., 2007). In this conformation, Y105 can form a T‐stacking interaction with bound cephalexin (Xiao et al., 2015), and the same is likely true for other commonly prescribed penicillins with aromatic moieties at similar positions (e.g., amoxicillin, phenoxymethylpenicillin, cefalexin). This T‐stacking interaction is likely critical for catalysis given that aromatic substitutions at this position have less of an impact on antibiotic resistance and enzyme kinetics than most other substitutions, per site‐saturation mutagenesis and kinetic analyses (Doucet et al., 2004). Importantly, this Y105 conformation is also more common in the FTA‐absent (*apo*) simulation than in the FTA‐bound (*holo*) simulation (43.7% vs. 34.6% of frames).

In the second Y105 side‐chain state, the phenolic ring is instead packed against P107. This conformation is more common in the FTA‐bound (*holo*) simulation than in the FTA‐absent (*apo*) simulation (29.5% vs. 11.8%). Y105 in this conformation cannot form a T‐stacking interaction with a penicillin bound in the orthosteric site. Further assessing whether these changes in dynamics directly impact catalysis (e.g., lactam ring hydrolysis) would require quantum mechanical calculations; however, it is reasonable to suppose that TEM‐1 is less able to recognize and catalyze orthosteric β‐lactam antibiotics when the stabilizing T‐stacking interaction is absent.

### 2.5 FTA may impact β‐lactam‐antibiotic binding via altered R244 dynamics

FTA binding was also associated with notable changes in R244 side‐chain conformational sampling (Figure 4b,c and Figure S3). In the initial *apo* TEM‐1 model (PDB 1ZG4; Stec et al., 2005), the R244 side chain points towards the orthosteric pocket. Crystal structures of TEM‐1 bound to orthosteric β‐lactam antibiotics (e.g., PDBs 1TEM (Maveyraud et al., 1996), 1JVJ (Wang et al., 2002), 1FQG (Strynadka et al., 1992), 1BT5 (Maveyraud et al., 1998)) show that R244 in this position plays a critical role in stabilizing the carboxy group common to many of these drugs (Yang et al., 2017; Zafaralla et al., 1992), and site‐directed mutagenesis has confirmed that R244 plays a role in catalysis (Zafaralla et al., 1992). Even though our *apo* simulations included no ligands, the R244 side chain remained in the penicillin‐amenable conformation 84.32% of the time.

In contrast, our initial *holo* (FTA‐bound) TEM‐1 model (based on PDB 1PZP; Horn & Shoichet, 2004) captured the R244 side chain in a conformation that would point away from the penicillin carboxyl group, in the general direction of the α12 C terminus (Figure 4b,c and Figure S3). Others have proposed that this alternate penicillin‐incompatible R244 conformation contributes to the underlying mechanism by which FTA inhibits TEM‐1 β‐lactamase activity (Horn & Shoichet, 2004; Olehnovics et al., 2021). As expected, the *holo* simulation sampled this initial conformation much of the time (16.5% of the *holo* simulation frames vs. 0.7% of the *apo* frames). Curiously, it also sampled the penicillin‐amenable conformation typical of the *apo* simulations (26.0% *holo* vs. 84.3% *apo*), as well as an intermediate conformation (37.2% *holo* vs. 3.0% *apo*). Calculated RMSF values confirm that FTA binding increases R244 dynamics; the RMSF of R244 was 79% higher in the FTA‐bound *holo* simulations (0.84 Å for *holo*, 0.47 Å for *apo*).

The arginine side chain contains four rotatable bonds, so the position of its positively charged terminal guanidine moiety in three‐dimensional space is not determined exclusively by its χ_1_ and χ_2_ angles. To directly measure this position relative to the orthorsteric penicillin‐binding site, we monitored the distances between the most distal arginine carbon (R244 Cζ) and the alpha carbon of an orthosteric‐pocket residue (S70 Cα) throughout the simulations (Figure 4d and Figure S4). We selected S70 Cα as the orthosteric‐site anchor because of the role it plays in the catalytic mechanism. In class A lactamases, β‐lactam ring opening occurs when E166, a general base, activates a catalytic water molecule that in turn activates S70, thus enabling nucleophilic attack on the β‐lactam carbonyl (Kalp et al., 2009; Matagne et al., 1998).

The distributions of these R244‐Cζ/S70‐Cα distance measurements agree with our *χ*_1_/*χ*_2_ assessment. The *apo* distance distribution is largely unimodal with a mode of 10.2 Å. In contrast, the *holo* distribution is bimodal. The first mode corresponds to that of the *apo* distribution (~10.2 Å), and the second is ~1.6 Å further from the pocket. FTA binding to TEM‐1 thus has a profound impact on R244 flexibility, allowing it to frequently adopt a penicillin‐incompatible conformation that precludes R244 stabilization of an orthosteric ligand.

Given that our simulations sample two distinct FTA poses (Figure 3) and that R244 is known to play a critical role in the allosteric mechanism (Horn & Shoichet, 2004), we were curious how R244 dynamics correlated with FTA orientation. We repeated the R244 distance analysis on the one *holo* simulation that captured the FTA‐pose transition, this time separately considering those frames with FTA in the vertical and horizontal poses, respectively. When the ligand is in the horizontal pose, the distance distribution has a single mode of 11.5 Å, the mode characteristic of the penicillin‐incompatible conformation (Figure S4). In contrast, the distance distribution when considering the vertical‐pose frames was still bimodal. These findings raise the possibility that the horizontal pose observed only in our simulations may be more effective at inhibiting TEM‐1 orthosteric activity than is the vertical pose observed crystallographically.

### 2.6 FTA impacts allosteric communication

Having identified the impact of FTA binding on specific residues, we next characterized the potential impact of FTA binding on allosteric communication. MD is an efficient way to elucidate allosteric pathways, especially when coupled with network analysis (Schueler‐Furman & Wodak, 2016; Yao & Hamelberg, 2022). Allostery can be seen as a transfer of information from one protein region (e.g., an allosteric site) to another (e.g., an orthosteric site).

To assess how critical a residue is for information transfer within the protein and how FTA impacts that transfer, we calculated the betweenness centrality (BC) of each residue (Amitai et al., 2004; del Sol et al., 2006; De Ruvo et al., 2012; Whitley & Lee, 2009; Basith et al., 2019). In brief, given a network of nodes connected by edges, BC measures how important a given node is for communication within the network. One first calculates all shortest paths (along the edges) between every node pair. The BC of a given node is determined by the number of shortest paths that pass through that node. In the context of a protein, the nodes are residues, and the edges connect residues near each other in 3D space (see Section 4 for details). Others have shown that residues implicated in allosteric and orthosteric mechanisms often have high centralities (Amitai et al., 2004; del Sol et al., 2006; De Ruvo et al., 2012; Whitley & Lee, 2009; Basith et al., 2019).

Four residues in the catalytic pocket (S70, K73, E166, K234) have lower BC values in the FTA‐bound *holo* simulations (Figure S5). In the *apo* protein, these residues play a larger role in controlling the flow of information. FTA‐binding reduces that role, perhaps explaining its impact on TEM‐1 catalysis. Another notable difference is the higher *holo*‐simulation BC values for residues at the β4 N‐terminus, including R244, which tends to be further from the catalytic pocket in the *holo* simulations than in the *apo* simulations (see above).

## 3 CONCLUSIONS

In this work, we used microsecond molecular dynamics simulations to study the allosteric mechanism whereby FTA inhibits TEM‐1 β‐lactamase activity. FTA binds ~15 Å from the orthosteric pocket, yet its binding has profound effects on protein dynamics. Even though *apo* TEM‐1 is a rigid protein, FTA binding further increases rigidity on average, as measured using RMSF analysis. Studies of TEM‐1 binding to the protein inhibitor BLIP have found similar increases in rigidity (Huang et al., 2020; Meneksedag et al., 2013), though there is some debate (Galdadas et al., 2021).

As others have noted (Horn & Shoichet, 2004; Olehnovics et al., 2021), FTA's allosteric mechanism is mediated largely through changes in R244 dynamics. In our *apo* simulations, R244 mostly samples a single rotomeric conformation. In contrast, in the *holo* simulations it samples at least two conformations, and one of those precludes key interactions between R244 and orthosteric‐bound penicillin observed crystalographically (e.g., PDB 1FQG; Strynadka et al., 1992). Y105, an amino acid that lines the catalytic pocket, also undergoes FTA‐dependent changes in sidechain dynamics that may impact penicillin binding. Finally, our simulations show that FTA changes intra‐protein communication between TEM‐1 residues, per BC calculations. These changes impact residues that line the catalytic pocket and so could also contribute to the allosteric mechanism.

Importantly, our simulations reveal a possible novel FTA binding pose that has never been observed crystallographically. In one of the three *holo* simulations, FTA underwent substantial rearrangement, burying itself between α11 and α12. We call this inserted pose the “horizontal” pose, in contrast to the “vertical” pose observed crystallographically (PDB 1PZP; Horn & Shoichet, 2004). Though the horizontal pose has never been observed experimentally, several lines of evidence support its existence. (1) In the 1PZP structure, the crystallographic FTA (vertical orientation) forms contacts with the neighboring protein in the crystal lattice, which may artefactually impact its binding mode. (2) The crystallographic pose of a different allosteric ligand (CBT, PDB 1PZO; Horn & Shoichet, 2004) positions a tetrazole moiety at a similar location as the horizontal‐pose FTA tetrazole. (3) A study performed by others using elastic network models predicted an open‐pocket conformation similar to that observed when FTA is in the horizontal pose (Kaynak et al., 2020), and studies using MD simulations and Markov state models confirm that the allosteric pocket may be larger than even ligand‐bound crystal structures suggest (Porter et al., 2019; Bowman & Geissler, 2012). (4) Induced‐fit docking suggests the horizontal and vertical FTA poses are similarly plausible. (5) MM‐GBSA suggests the horizontal pose is more energetically favorable than the vertical pose. (6) Binding pose metadynamics suggests the horizontal pose is more stable than the vertical pose. (7) The horizontal pose better locks R244 into a penicillin‐incompatible conformation than does the vertical pose.

This work is significant because the TEM‐1/FTA complex is frequently used as a model protein to study allostery (Meneksedag et al., 2013; Galdadas et al., 2021; Porter et al., 2019; Bowman & Geissler, 2012; Bowman et al., 2015; Modi & Ozkan, 2018; Knoverek et al., 2021; Modi et al., 2021), and the horizontal FTA pose observed in our simulations challenges our understanding of the allosteric mechanism. It is, of course, possible that the predicted horizontal pose is artefactual, a consequence of the inaccuracies inherent in MD force fields. Alternatively, the crystallographic FTA pose may be a legitimate metastable state along the binding pathway from bulk solvent to the horizontal‐pose end point. But the possibility of an alternate FTA pose also serves as a cautionary tale. While crystallography forms the basis of much of computational structural biology, it is not infallible. Crystal contacts can capture ligand poses that may not be physiological, especially when soaking is used rather than co‐crystallization. We are hopeful that this new insight will further drug discovery targeting the allosteric pocket of β lactamases.

## 4 MATERIALS AND METHODS

### 4.1 TEM‐1 modeling

We modeled *apo* TEM‐1 based on the unmodified 1ZG4 (Stec et al., 2005) structure. In contrast, we modeled the *holo* system by modifying the 1PZP (Horn & Shoichet, 2004) structure. This structure differs from 1ZG4 by three amino acids (I84V, N100R, and V184A), so we used ChimeraX (Pettersen et al., 2021) to change the 1PZP residues so the sequences would be identical. The 1PZP structure also includes two copies of the small‐molecule inhibitor 3‐(4‐phenylamino‐phenylamino)‐2‐(1H‐tetrazol‐5‐yl)‐acrylonitrile (FTA). Visual inspection of the structure, electron density maps, and crystal contacts showed that the first FTA ligand binds in the cryptic, allosteric pocket. The second is completely embedded at a protein–protein interface, a product of crystallographic packing. We removed the second FTA ligand because it is unlikely to be physiologically relevant. After making these changes, the only differences between the two systems were the atomic coordinates of protein atoms and the presence of the FTA ligand. For completeness’ sake, we also modeled a third system in which the *holo* (1PZP) system was not subjected to computational mutagenesis. To simplify the discussion and presentation of results, we describe this third system in Appendix S1.

MolProbity (Williams et al., 2018) applied to both the *apo* and *holo* proteins suggested we flip residues Q39 and N276 to optimize the hydrogen‐bond network. We added hydrogen atoms to all protein residues using the PDB2PQR (Jurrus et al., 2018) web server, which uses the PROPKA (Li et al., 2005) algorithm (pH 7). We added hydrogen atoms to the FTA ligand using the LEaP reduce function (part of AmberTools20, default parameters) (Case et al., 2020) and assigned partial charges to the ligand atoms using the Antechamber package at the AM1‐BCC level of theory (default parameters).

We solvated each system with an enveloping water box (10 Å padding) after rotating each to minimize the required box volume. We neutralized all systems using Na^+^ counter ions and then added Na^+^ and Cl^−^ ions as required to achieve a 0.15 M solution. The systems were parameterized in Antechamber using the FF14SB (Maier et al., 2015), GAFF (Wang et al., 2004), and TIP3P (Jorgensen et al., 1983) force fields for protein, ligand, and water residues, respectively.

### 4.2 Molecular dynamics simulations

We performed molecular dynamics (MD) simulations using the NAMD 2.13 package (Phillips et al., 2020). The systems were minimized via a four‐step process of 5000 steps each. First, all hydrogen atoms were relaxed; then hydrogen atoms and water molecules; then hydrogen atoms, water molecules, and protein side chains; and finally, all atoms.

We equilibrated each system under NPT (isothermal–isobaric) conditions at 310 K using long‐range Particle Mesh Ewald electrostatics with Langevin dynamics (damping constant 5/ps). For the *apo* simulations, we performed a four‐step equilibration. In each 0.25 ns step, we applied harmonic constraints to the protein backbone atoms, which we gradually relaxed at each step (1.0, 0.75, 0.5, 0.25 kcal/mol/Å^2^, respectively). For the *holo* simulations, we performed a single 1 ns equilibration step with a 1 fs time step.

After equilibration, we set up three production runs per system (250 ns, 250 ns, and 500 ns) with identical conditions but different random seeds. To determine if the system was equilibrated, we calculated the backbone RMSD between each simulated frame and the first frame using MDAnalysis (V1.0.0) (Gowers et al., 2016; Michaud‐Agrawal et al., 2011). This analysis suggested that the systems continued to equilibrate through the first 10 ns of the production runs (Figure S1). We trimmed the initial 10 ns of each run for consistency and used these truncated runs for all remaining analyses. Finally, we aligned the simulations by their backbone atoms using MDAnalysis.

### 4.3 Induced fit docking, MM‐GBSA, and binding pose metadynamics

In one of the *holo* simulations, the FTA ligand shifted from the crystallographic (“vertical”) pose to a “horizontal” pose (see Section 2). To generate a representative conformational ensemble of the protein/ligand system, we first performed two agglomerative hierarchical clustering analyses focused on the vertical‐ and horizontal‐pose portions of this simulation, respectively, and identified the centroid of the most populated cluster in each case. To this ensemble of two conformations, we added the original crystallographic (1PZP; Horn & Shoichet, 2004) conformation (vertical pose) and a hand‐selected simulation frame that captured the ligand in the horizontal pose. We used Schrödinger's Protein Preparation Wizard to further process each of these four conformations for subsequent studies.

To prepare the FTA ligand for docking, we used Schrödinger LigPrep (2021) with the OPLS4 forcefield (default parameters). We then used the Schrödinger induced‐fit docking (Sherman et al., 2006a, 2006b) (IFD) module with extended sampling to re‐dock FTA into the four protein conformations (default parameters). To calculate binding free energies, we used Prime‐MMGBSA to reassess the 15 best‐scoring poses associated with the vertical and horizontal poses, respectively. Finally, we used Schrödinger's binding pose metadynamics (BPMD) module (Clark et al., 2016) to assess the stability of the lowest‐binding‐energy (per MMGBSA) vertical and horizontal poses. For comparison's sake, we similarly applied BPMD to the vertical crystallographic pose (1PZP; Horn & Shoichet, 2004) and the hand‐selected “horizontal” pose, independent of IFD/MMGBSA.

### 4.4 Analysis of MD simulations

We used MDAnalysis (Gowers et al., 2016; Michaud‐Agrawal et al., 2011) and CPPTRAJ (Roe et al., 2013) to calculate RMSD, RMSF, and atomic‐distance values, and to cluster the protein conformations. We calculated and plotted side‐chain angles for select residues using the Janin class of MDAnalysis. K‐Means clustering of Janin plots was performed using Scikit‐Learn (Pedregosa et al., 2011). To predict TEM‐1 secondary structure, we used the online server Stride (Frishman & Argos, 1995) with PDB 1ZG4 (Stec et al., 2005) as the template. To assess the distance between R244‐Cζ and S70‐Cα, we fit atomic‐distance distributions to bimodal distributions using the *curve_fit* function available in SciPy (Virtanen et al., 2020).

### 4.5 Network analysis

We used MD‐TASK (Brown et al., 2017) to calculate the betweenness centrality (BC) of each protein residue. In brief, we first constructed a network comprised of nodes—Cβ atoms or Cα for glycine. We connected all nodes within 7.0 Å of each other with edges and calculated the shortest paths (in network space) between all node pairs. The BC of a given node is the number of these shortest paths that pass through that node. BC essentially measures how important a given node is for communication within the network (protein). To assess the impact of FTA binding, we considered changes in BC across the trajectories. Residues with distinctly different behavior in the *apo* and *holo* simulations (three standard deviations above the mean) were considered large changes.

## AUTHOR CONTRIBUTIONS

**Erich Hellemann:** Conceptualization (equal); data curation (lead); formal analysis (lead); investigation (lead); methodology (equal); software (lead); supervision (supporting); visualization (equal); writing – original draft (equal); writing – review and editing (equal). **Amrita Nallathambi:** Formal analysis (supporting); investigation (supporting); writing – original draft (supporting); writing – review and editing (supporting).

## FUNDING INFORMATION

This work was supported by the National Institute of General Medical Sciences of the National Institutes of Health [R01GM132353 to Jacob D. Durrant]. The content is solely the responsibility of the authors and does not necessarily represent the official views of the National Institutes of Health.

## CONFLICT OF INTEREST STATEMENT

The authors have no conflict of interest to declare.

## Supporting information


**Appendix S1:** Supporting Information

**Table S1.** Center‐of‐geometry RMSF per residue for all the simulations. The *holo_WT* simulations are the FTA‐bound simulations described in the main text. The *apo_WT* simulations are the *apo* simulations described in the main text, which have the same amino‐acid sequence (S#864) as the *holo_WT* simulations. The *holo_mut* simulations (S#256) are the *holo* simulations described in the SI. Note that the ΔRMSF values presented in Figure 2 are given as the last column for reference. STD, standard deviation; SEM, standard error of the mean.

## Data Availability

The data that support the findings of this study are available from the corresponding author upon reasonable request.
