# Supplementary material for: Allosteric inhibition of TEM‐1 β lactamase: Microsecond molecular dynamics simulations provide mechanistic insights
Source: Protein Sci. 2023 Apr 1;32(4):e4622. doi: 10.1002/pro.4622 (PMC10044111; doi:10.1002/pro.4622)
Supplement: Supplementary file 1 — Appendix S1: Supporting Information [file PRO-32-e4622-s002.pdf]

SUPPORTING INFORMATION

Allosteric inhibition of TEM-1 β lactamase: microsecond molecular dynamics simulations provide mechanistic insights

The main text describes MD simulations of *apo* and *holo* (FTA-bound) TEM-1. The sequences of these two proteins are identical and correspond to entry S#864 in the Lactamase Engineering Database (LacED)[1] (i.e., the 1ZG4 crystal-structure sequence). We also simulated a third *holo* (FTA-bound) system whose sequence corresponded to LacED entry S#256 (i.e., the 1PZP crystal-structure sequence). The S#864 and S#256 sequences differ by only three amino acids, so the two *holo* simulations behaved similarly. To simplify the discussion and presentation of results, we described only the S#864 *holo* simulation in the main text. Here we additionally describe the S#256 *holo* simulation, which was generally similar to the S#864 *holo* simulation except it did not capture the horizontal FTA pose. For clarity's sake, we will refer to S#864 TEM-1, described in the main text, as wildtype (WT) TEM-1. We will refer to S#256 TEM-1 as mutant TEM-1.

Simulation equilibration

To determine how much the TEM-1 conformations changed over the course of the simulations, we aligned them and calculated the backbone-atom root-mean-square distances (RMSD) between each frame and the respective initial frame (Figure S1). The WT *apo*, WT *holo*, and mutant *holo* TEM-1 simulations are shown on the top, middle, and bottom rows, respectively.

We discarded the first 10 ns of each simulation (marked with a dashed vertical line) to ensure all simulations had properly equilibrated.

The WT *apo* simulations deviated more from the starting position ($\langle bbRMSD_{apo} \rangle = 1.15 \pm 0.17$ Å) than the WT *holo* simulations ($\langle bbRMSD_{holo\ WT} \rangle = 0.91 \pm 0.10$ Å), though the deviations were modest in both cases. These findings harmonize with previous nuclear magnetic resonance (NMR)[2] and molecular dynamics (MD) studies[3-5], which have shown that TEM-1 is a highly rigid protein with high order parameters ($\langle S^2 \rangle = 0.90 \pm 0.02$) and only minor conformational changes in simulations (per RMSD).

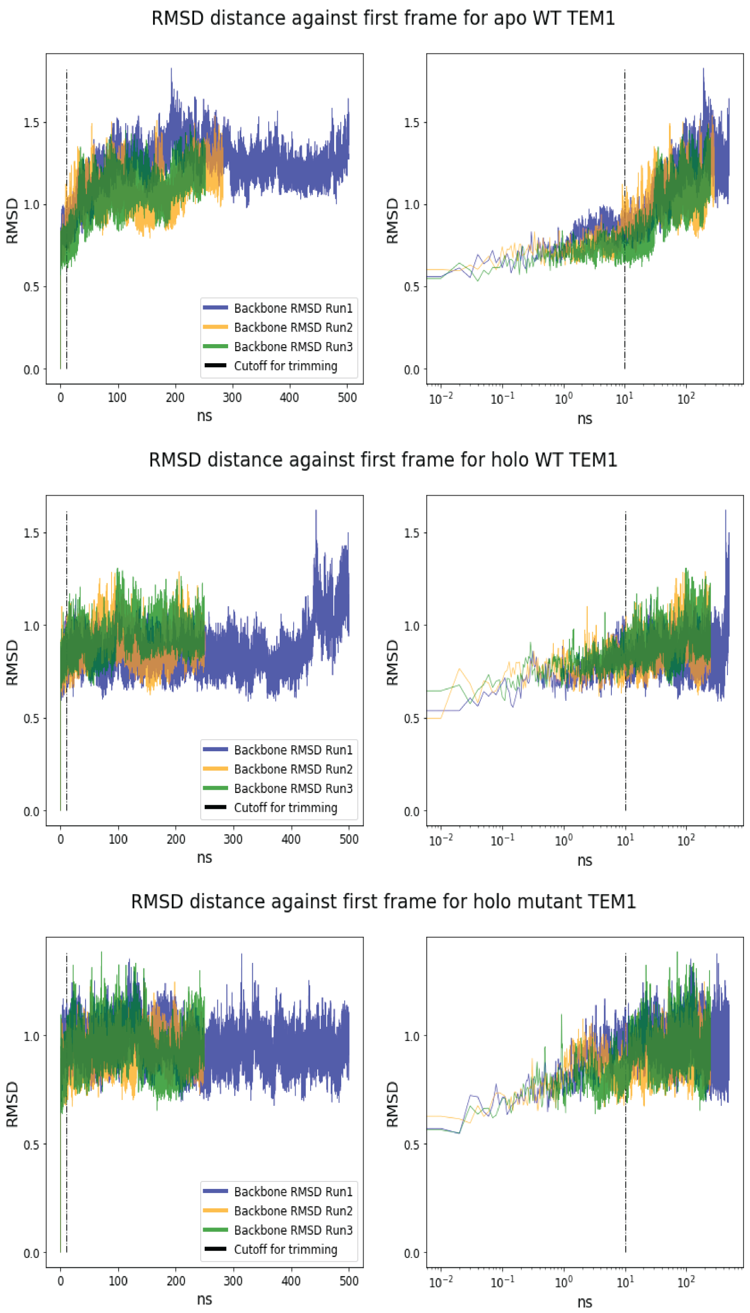

Figure S1. Plots of the TEM-1 backbone RMSD to the first frame. In the top row, the WT *apo* (S#864) simulations. In the middle row, the WT *holo* (S#864) simulations. In the third row, the mutant *holo* (S#256) simulations. The two graphs on each row are identical, except the second graph uses a logarithmic scale on the X axis.

FTA decreases TEM-1 flexibility

We calculated differences in per-residue RMSF values, *apo* - *holo*, for the mutant *holo* simulations, just as we did for the WT *holo* simulations (Figure S2). With few exceptions, the WT and mutant *holo* simulations differed similarly from the *apo* simulation.

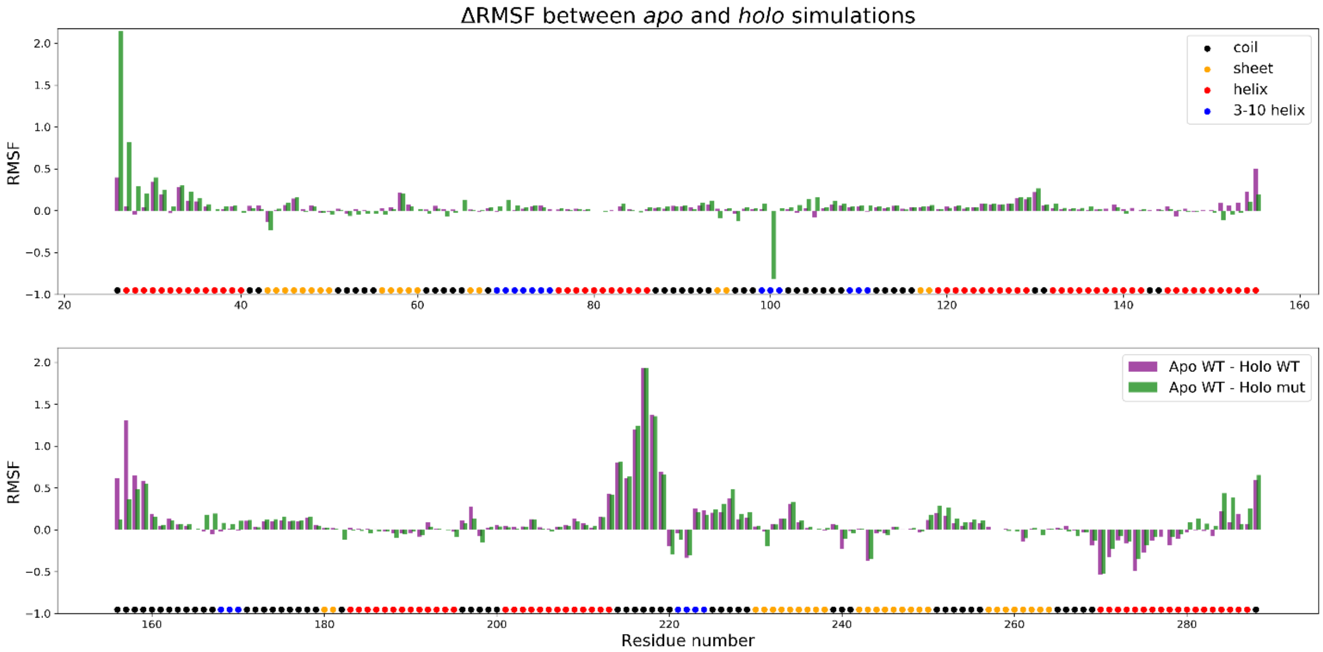

Figure S2. Differences in center-of-geometry RMSF per residue. RMSF differences between the two *holo* simulations are small (less than 0.3 Å), except for the coil after α7, where *holo* mutant residues are more flexible than *holo* WT residues. There is one outlier according to COG RMSF: residue 100. This residue is one of the mutations observed in 1PZP (N100R), but if we consider the RMSF of only Cα atoms, the *holo* simulatios differ by only 0.026 Å. Catalytic residue S70 and the general base E166 have lower RMSF values for the *holo* mutant simulations, but the changes fall below 0.3 Å.

FTA impact on selected residues

In the main text, we provide Janin plots for Y105 and R244, WT *apo* vs. WT *holo* (Figure 4 A and 4B). We did not include Janin plots for other residues implicated in ligand recognition, ligand stabilization, and catalysis because FTA binding did not have a substantial impact on their side-chain dynamics. But we include these plots here for completeness' sake, together with Janin plots for the mutant *holo* simulations (Figure S3).

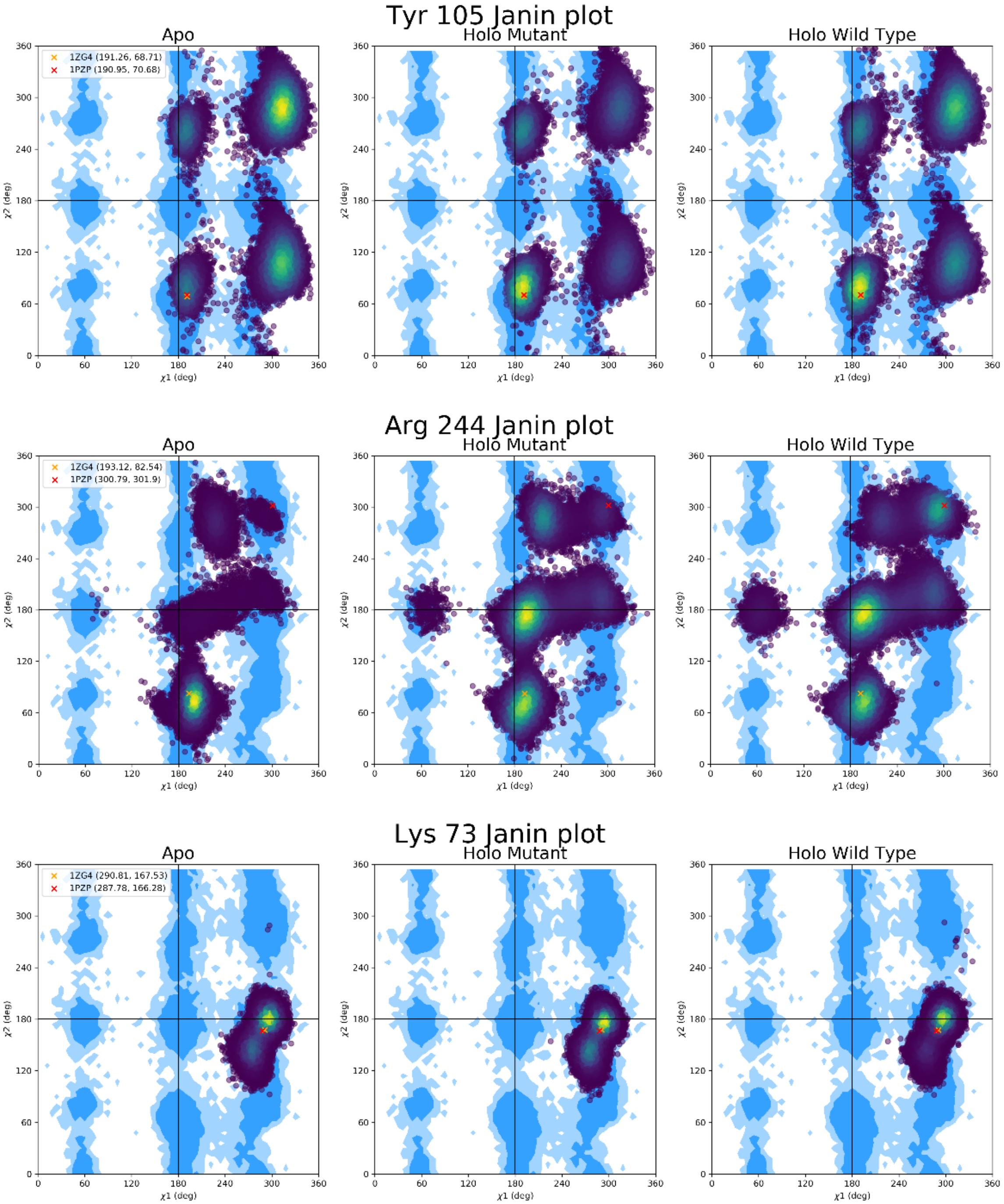

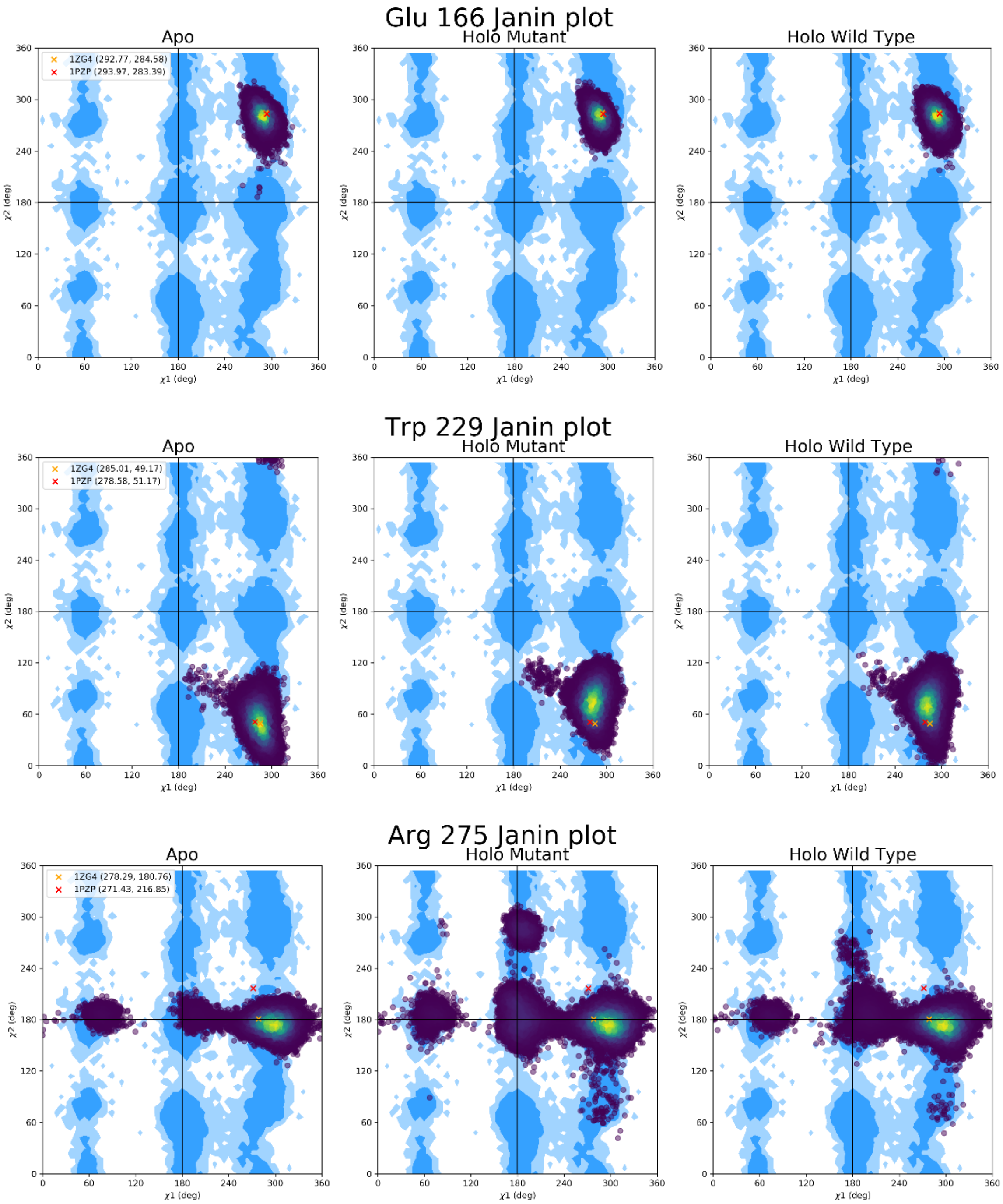

Figure S3. Janin plots of select residues, *apo* simulation (left), *holo* mutant simulation (middle), and *holo* WT simulation (right).

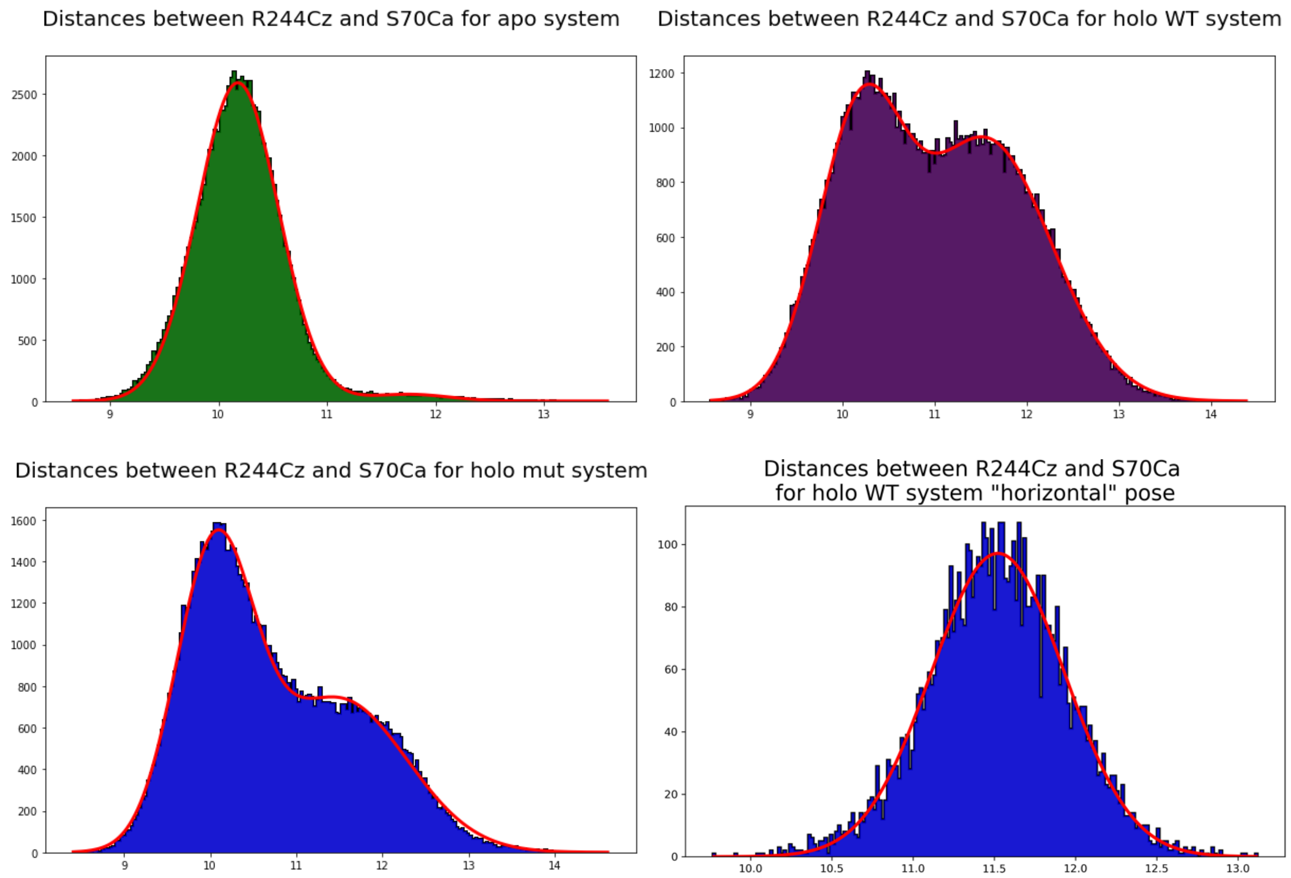

Figure S4. Distributions of the distance between R244 Cζ and S70 Cα. Top left, the WT *apo* simulation. Top right, the WT *holo* simulation. Bottom left, the mutant *holo* simulation. Bottom right, considering only the portion of the WT *holo* simulation with the ligand in the horizontal pose.

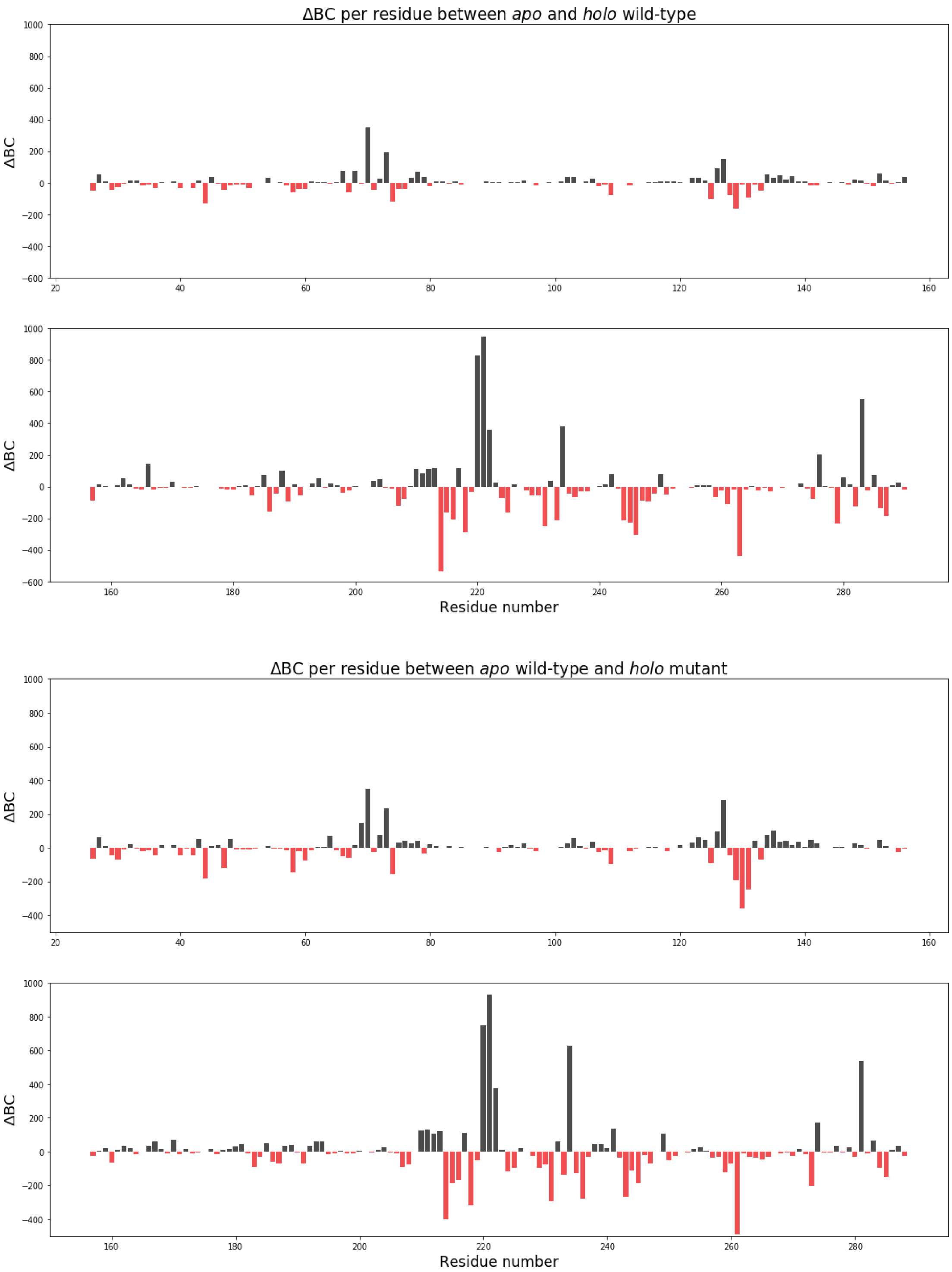

Figure S5. *Apo* vs. *holo* differences in BC, per residue, WT (S#864) TEM-1 and mutant (S#256) TEM-1. Four residues in the catalytic pocket (S70, K73, E166, K234) have lower BC values in the FTA-bound *holo* simulations than in the *apo* simulations. Additionally, residues at the β4 N-terminus have higher BC values in the *holo* simulations than in the *apo* simulations. This includes R244, which tends to be further from the catalytic pocket in the *holo* simulations. We note that large BC differences in the α11 and α12 helices (A217-A224 and M272-H289) are likely artefactual; when FTA is present (*holo*), the gap it forms decreases the BC for some residues in these helixes.

References

1 Thai, Q. K., Bos, F. & Pleiss, J. The Lactamase Engineering Database: a critical survey of TEM sequences in public databases. *BMC Genomics* **10**, 390, doi:10.1186/1471-2164-10-390 (2009).

2 Savard, P. Y. & Gagne, S. M. Backbone dynamics of TEM-1 determined by NMR: evidence for a highly ordered protein. *Biochemistry* **45**, 11414-11424, doi:10.1021/bi060414q (2006).

3 Roccatano, D. *et al.* Dynamical aspects of TEM-1 beta-lactamase probed by molecular dynamics. *J Comput Aided Mol Des* **19**, 329-340, doi:10.1007/s10822-005-7003-0 (2005).

4 Bös, F. & Pleiss, J. Multiple molecular dynamics simulations of TEM β-lactamase: Dynamics and water binding of the Ω-loop. *Biophys J* **97**, 2550-2558 (2009).

5 Giampaolo, A. D. *et al.* On the structural affinity of macromolecules with different biological properties: molecular dynamics simulations of a series of TEM-1 mutants. *Biochem Biophys Res Commun* **436**, 666-671, doi:10.1016/j.bbrc.2013.06.013 (2013).
